# Leptospiral adhesins: from identification to future perspectives

**DOI:** 10.3389/fmicb.2024.1458655

**Published:** 2024-08-13

**Authors:** Matthew C. Surdel, Jenifer Coburn

**Affiliations:** ^1^Division of Infectious Diseases, Department of Medicine, Medical College of Wisconsin, Milwaukee, WI, United States; ^2^Department of Microbiology and Immunology, Medical College of Wisconsin, Milwaukee, WI, United States

**Keywords:** *Leptospira*, leptospirosis, adhesion, adhesins, binding, spirochete

## Abstract

Leptospirosis is a significant zoonosis worldwide, with disease severity ranging from a mild non-specific illness to multi-organ dysfunction and hemorrhage. The disease is caused by pathogenic bacteria of the genus *Leptospira*, which are classified into pathogenic and saprophytic clades. Bacterial binding to host molecules and cells, coordinated by adhesin proteins, is an important step in pathogenesis. While many leptospiral adhesins have been identified, the vast majority have not been characterized *in vivo*. Herein, we present an overview of the current methodologies and successes in identifying adhesins in *Leptospira*, including known biological roles *in vivo*. We will also identify and discuss potential areas for future research.

## 1 Introduction

Leptospirosis, the most widespread zoonosis globally, is caused by gram-negative spirochetes of the genus *Leptospira* (Costa et al., 2015; European Centre for Disease Prevention and Control, 2017). Disease ranges from a non-specific febrile illness to multiorgan dysfunction and hemorrhage, causing significant morbidity and mortality worldwide with the greatest impact on resource-poor populations (Weil, 1886; Alston and Brown, 1937; Gouveia et al., 2008; Costa et al., 2015). Leptospirosis is estimated to cause 60,000 deaths and a loss of productivity of $50 billion annually (Costa et al., 2015; Torgerson et al., 2015; Agampodi et al., 2023). Leptospirosis also affects animals, both domestic and wild, leading to substantial costs to the agricultural industry (Matthews et al., 1987; Barwick et al., 1998; Bennett et al., 1999; Ramos et al., 2006; Lilenbaum et al., 2008; Miotto et al., 2018; Canton et al., 2022).

*Leptospira* spp. are categorized into more than 300 serovars (serological variants) based upon sera reactivity to cell surface antigens, and into two clades (pathogenic, P, and saprophytic, S) and four subclades (P1, P2, S1, and S2) based upon genomic sequencing (Thibeaux et al., 2018; Vincent et al., 2019). *Leptospira* species range from highly pathogenic organisms in the P1 subclade, to free-living saprophytes unable to infect hosts in the S clade (Vincent et al., 2019). The enzootic cycle of *Leptospira* involves colonization of the renal proximal tubules in reservoir hosts, followed by shedding in the urine and infection of new hosts; humans are incidental hosts (reviewed in Adler and de la Pena, 2010; Adler et al., 2014; Haake and Levett, 2015).

*Leptospira* spp. remain significantly understudied. To combat this disease, researchers must determine how the pathogen is able to adapt and survive in various hosts and environments, which likely requires an interplay of bacterial, host, and environmental factors. One step in the infection process is binding to host tissue components. Bacteria produce a wide range of proteins known as adhesins that are responsible for binding host cells and molecules (reviewed in Marra and Isberg, 1996; Niemann et al., 2004; Pizarro-Cerda and Cossart, 2006; Patel et al., 2017; Paulsson and Riesbeck, 2018; Daroz et al., 2021). The specific adhesins produced by an organism contribute to its tropism and allow for successful infection. Numerous leptospiral outer membrane proteins (OMPs) have been shown to bind host cells and molecules, but the role the candidate adhesins play in natural infection remains unclear. A major question in the field remains: what is the role of these adhesins during initial contact with the host, and how do they contribute to colonization and adhesion to the proximal tubules of the kidneys? A detailed understanding of the arsenal of adhesins used by *Leptospira* is needed. Understanding this complex process will set the foundation for development of novel vaccines and therapeutic strategies to combat leptospirosis.

**Figure 1:**
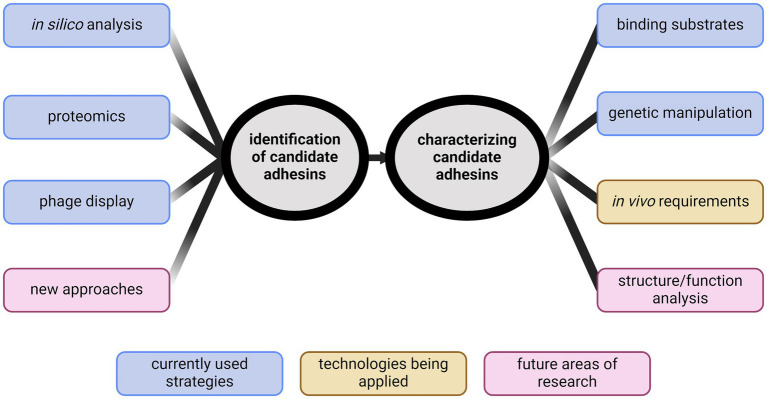
Identification of new directions for studying leptospiral adhesins. Identifying adhesins has primarily been performed through *in silico* analyses, phage display, or proteomics. New ideas for identification are needed. Characterization of candidate adhesins has typically been through demonstrating binding. Recent advancements in genetic manipulation of *Leptospira* spp. have provided new abilities to characterize candidates *in vivo*. Future research will require developing novel methods to identify biologically relevant adhesins and begin structure/function analyses to further define adhesin function. Created with BioRender.com.

The goal of this review is to summarize methods of adhesin identification, highlight the gaps in knowledge regarding adhesins, and discuss what is needed in future adhesin research in *Leptospira* (Figure 1).

## 2 Identification of *Leptospira* adhesins

### 2.1 The era of genomics

Whole genome sequencing revolutionized bacterial research. The first two bacterial genomes sequenced were those of *Haemophilus influenzae* and *Mycoplasma genitalium* in 1995 (Fleischmann et al., 1995; Fraser et al., 1995). By 2000, approximately 23 bacterial genomes had been described, with many more soon to follow (Schwartz, 2000). The first full *Leptospira* genome, *L. interrogans* serovar Lai, was reported by Ren et al. (2003). The genome consisted of two chromosomes of 4.33 megabases and 359 kilobases, encoding a total of 4,768 predicted genes (Ren et al., 2003). In 2004, the genome of *L. interrogans* sv. Copenhageni was described (Nascimento et al., 2004a,b). The non-pathogenic isolate *L. biflexa* sv. Patoc genome sequence was published by Picardeau et al. (2008). Pathogenic strains of *Leptospira* bind to human cells and ECM components more efficiently than do avirulent or saprophytic (S1) leptospires (Tsuchimoto et al., 1984; Ito and Yanagawa, 1987; Merien et al., 1997; Evangelista K. et al., 2014; Evangelista K. V. et al., 2014). This set the stage for identifying potential adhesins through comparative *in silico* analysis.

The program SpLip was designed to predict potential surface exposed lipoproteins that was based upon experimental data (Nascimento et al., 2004a; Setubal et al., 2006). Proteins predicted to be surface exposed were compared among the pathogenic isolates *L. interrogans* svv. Lai and Copenhageni (Ren et al., 2003; Nascimento et al., 2004a,b). Proteins of interest were screened for adhesive function, forming the basis for the identification and characterization of over 40 candidate adhesins by Nascimento et al. (2004a,b) (Gamberini et al., 2005; Barbosa et al., 2006; Vieira et al., 2007, 2010a,b; Atzingen et al., 2008, 2009; Gomez et al., 2008; Longhi et al., 2009; Oliveira et al., 2010, 2011, 2013; Mendes et al., 2011; Domingos et al., 2012, 2015; Fernandes et al., 2012, 2014; Souza et al., 2012; Siqueira et al., 2013, 2016, 2017; Silva et al., 2016; Pereira et al., 2017; Santos et al., 2018; Cavenague et al., 2019, 2023; Kochi et al., 2019, 2020; Rossini et al., 2020; Passalia et al., 2020a,b, 2021; Takahashi et al., 2021, 2022). Many of these proteins interact with multiple host substrates, often binding the same substrates as other candidates (Nascimento et al., 2004a,b; Gamberini et al., 2005; Barbosa et al., 2006; Vieira et al., 2007, 2010a,b; Atzingen et al., 2008, 2009; Gomez et al., 2008; Longhi et al., 2009; Oliveira et al., 2010, 2011, 2013; Mendes et al., 2011; Domingos et al., 2012, 2015; Fernandes et al., 2012, 2014; Souza et al., 2012; Siqueira et al., 2013, 2016, 2017; Silva et al., 2016; Pereira et al., 2017; Santos et al., 2018; Cavenague et al., 2019, 2023; Kochi et al., 2019, 2020; Rossini et al., 2020; Passalia et al., 2020a,b, 2021; Takahashi et al., 2021, 2022). It must be noted that non-specific binding *in vitro* cannot be ruled out. Taken together, this research suggests that *Leptospira* likely interact with numerous host proteins throughout infection and indicates that there is significant redundancy in the leptospiral adhesin proteome. The wide binding and redundancy likely aid *Leptospira* during infection, but also complicates research. Loss of a single adhesin is therefore less likely to cause a significant virulence defect, meaning therapeutic approaches targeting adhesion will need to include multiple proteins.

*In silico* analysis was also performed to identify genes in *L. interrogans* that had homology to known adhesin genes from other species. Proteins containing leucine-rich repeat (LRR) domains from other bacterial pathogens interact with host cells (Bierne et al., 2007; Ng and Xavier, 2011). Pathogenic *Leptospira* encode significantly more LRR proteins than do non-pathogenic *Leptospira*; LIC10831, an LRR protein from *L. interrogans*, was investigated and binds human cadherins (Eshghi et al., 2015; Fouts et al., 2016). In addition to comparisons between species, comparisons within a single genome have also identified adhesive proteins. LenA, also known as Lsa24 and LfhA, was known to have adhesive function (Barbosa et al., 2006). Stevenson et al. (2007) searched the *L. interrogans* genome for homology to *lenA* and identified five paralogs which they designated *lenB*-*F*. All Len proteins interact with laminin, but the newly identified paralogs also bind fibronectin, which can also be a component of the extracellular matrix (Barbosa et al., 2006; Stevenson et al., 2007).

Adhesins have also been identified in other *Leptospira* species. *L. borgpetersenii* is a species commonly carried in dairy cattle around the world (Hamond et al., 2022; Mazzanti et al., 2023), but can infect humans (Slack et al., 2006; Bourhy et al., 2012; Rajaonarivelo et al., 2023). Kamaruzaman et al. (2024) recently published an *in silico* analysis of *L. borgpetersenii* sv. Hardjo-Bovis looking for predicted surface exposed proteins present only in pathogens. They identified LBL0972 and LBL2618 that have homologs in the *L. interrogans* genome, and bind multiple substrates, including fibronectin, laminin, and fibrinogen (Kamaruzaman et al., 2024). A number of other candidate adhesins were identified from additional *in silico* approaches, including but not limited to LcpA (Barbosa et al., 2010; da Silva et al., 2015), LIC11966 (Ghosh et al., 2019), OmpL37 (Pinne and Haake, 2009; Pinne et al., 2010), MPL36 (Vieira et al., 2010a; Zhu et al., 2023), and LIC10271 (Sarma et al., 2023).

Taken together, *in silico* genomic analyses have been successful in identifying candidate adhesins. Today there are over 60 species and 300 serovars of *Leptospira* identified, with 72 reference genomes deposited in NCBI (Genome, 1988-2024; Thibeaux et al., 2018; Casanovas-Massana et al., 2019; Vincent et al., 2019; reviewed in Caimi and Ruybal, 2020; Dos Santos et al., 2023). This wealth of data will allow for extensive comparative analysis, helping to categorize strains by virulence and identify key differences. However, the *in vivo* role of the majority of the candidate adhesins is unknown. An additional complication is that many *Leptospira* adhesins bind extracellular matrix components, but specificity in biologically relevant conditions remains to be demonstrated. Future research must distinguish proteins that bind components *in vitro* from those with a biological and pathogenic role *in vivo*. Characterizing adhesins with *in vivo* roles will drive the development of future vaccine and therapeutic approaches.

### 2.2 Proteomics

As genomics advanced, so did proteomics. Pinne et al. (2012) combined genomics and proteomics to identify novel adhesins from *L. interrogans* sv. Copenhageni that bound fibronectin. *In silico* analysis was performed to determine a large set of genes that are predicted to encode surface proteins. A microarray containing these proteins was created and probed with host molecules to identify host-bacterial interactions. This method identified 15 fibronectin binding proteins, and Pinne et al. (2012) were able to confirm LIC10258, LIC10537, LIC10714, LIC11051, LIC11436, LIC11612, and LIC12631 bind fibronectin *in vitro* (Pinne et al., 2012).

Instead of predicting proteins on the outer surface, some groups have directly identified them on the basis of experimental data. One example is the leptospiral immunoglobulin-like proteins (Ligs), initially identified by searching for *L. interrogans* sv. Pomona proteins that reacted with sera from infected animals (Palaniappan et al., 2002). This study aimed to identify surface-exposed proteins to use as vaccine candidates, rather than identifying adhesins specifically. Lig proteins also contain domains similar to those of *Y. pseudotuberculosis* invasin and *E. coli* intimin, two known adhesins, thus suggesting the Lig proteins could play a role in *Leptospira* adhesion (Matsunaga et al., 2003). Later, other groups further characterized this three-protein family and confirmed the Lig proteins function as adhesins that bind laminin and fibrinogen (Choy et al., 2007; Castiblanco-Valencia et al., 2012; Hsieh et al., 2016). Lig proteins were of special interest because they were produced during infection, as evidenced by the host immune response, and had a known function. Despite efforts to develop a vaccine using recombinant Lig proteins, success has been limited. Multiple studies have shown that immunizing with one or a combination of Lig proteins, while protective against death, did not prevent infection and colonization (Palaniappan et al., 2006; Silva et al., 2007; Coutinho et al., 2011; Evangelista et al., 2017). Leptospires could still be shed and infect new hosts.

LipL32 was initially identified as an OMP produced during infection (Haake et al., 2000). Haake et al. (2000) identified the most prominent protein band in *L. kirschneri* by SDS-PAGE and, through further purification and sequencing, identified the *lipL32* gene. Homologs of *lipL32* were identified in pathogenic *Leptospira* but were not identified in non-pathogens (Haake et al., 2000). Later, LipL32 was confirmed by independent methods to be the most abundant protein on the outer membrane (Cullen et al., 2005; Malmstrom et al., 2009). It was subsequently characterized as an adhesin that binds fibronectin, collagens, laminin and human umbilical vein endothelial cells (HUVECs) (Hauk et al., 2008; Hoke et al., 2008; Sun et al., 2010). Thus, focusing on confirmed OMPs has also provided valuable information on candidate adhesins in *Leptospira*.

### 2.3 Phage display

Phage display, first reported by Smith (1985) as a method for purifying antibodies based upon affinity, has evolved significantly. A notable iteration is shotgun phage display, where random genome fragments are inserted into a phage coat gene, and therefore are present on the surface of the phage (Jacobsson et al., 2003). The resulting library is screened for fragments encoding peptides that bind to a chosen substrate. Enrichment of phage is performed multiple times in succession, and selected clones are isolated and sequenced to identify the encoded gene fragment. There are many advantages of using phage display: (1) adhesin identification relies on functional assays rather than predictions from *in silico* analysis, (2) as protein fragments are being analyzed, functional data is collected regarding specific domains and residues that are important in adhesion, and (3) identifying similar overlapping fragments increases the specificity and accuracy of data collected.

Lima et al. (2013) performed a shotgun phage display experiment *in vitro* and *in vivo* to identify proteins that bind kidney cells by creating a library from fragmented *L. interrogans* sv. Copenhageni DNA. Phage enrichment was performed based upon adherence to pig renal epithelial cells *in vitro*, or *in vivo* through injection into the heart of hamsters where phage was allowed to circulate for 5 min before harvesting the kidneys (Lima et al., 2013). LIC12976 was identified by both *in vitro* and *in vivo* strategies (Lima et al., 2013). To our knowledge, this is the only experiment identifying proteins based on function in a living host, and therefore provides clear biological relevance. An additional study by the same group enriched for phage that bound monkey renal epithelial cells (Lauretti-Ferreira et al., 2022). LIC10778 was identified to bind laminin, collagens I and IV, vitronectin and plasma, and cell fibronectins (Lauretti-Ferreira et al., 2022). The importance of LIC10778 *in vivo* remains unknown.

Another group identified proteins from *L. interrogans* sv. Copenhageni by phage display that bind Ea.hy926 endothelial cells *in vitro* (Evangelista et al., 2014). Evangelista K. V. et al. (2014) characterized LIC11574 and LIC13411 as candidate adhesins that bind VE-cadherin with nanomolar affinities and also bind other cadherins. Later work has followed up on this observation and demonstrated that heterologous production of LIC13411 increased binding of a non-pathogen to host tissues, which will be discussed in the next section of this review (Surdel et al., 2022b). Taken together, phage display is a valuable method for identifying candidate host-binding proteins in bacteria. The *Leptospira* field would greatly benefit from expanding this technique to identify additional biologically relevant adhesins *in vivo*. For example, *in vivo* phage display was used to find candidate *Borrelia burgdorferi* adhesins that bind the endothelium (Antonara et al., 2007). The known integrin-binding adhesin P66 was identified (Coburn et al., 1999; Coburn and Cugini, 2003; Kumar et al., 2015), as well as many novel adhesins that have all been validated by subsequent work (Antonara et al., 2007; Verma et al., 2009; Lin et al., 2015; Yang et al., 2016; Lin et al., 2020). Adapting this method to study *Leptospira* could provide novel insight into the arsenal of adhesins important during infection.

## 3 Expanding research beyond identification

Genomics, proteomics, and phage display have led to the identification of countless leptospiral candidate adhesins. As highlighted by this review, many lack functional data in a living host. To fully understand *Leptospira* pathogenesis, the field needs to move beyond identification and describe the function of these proteins in a living bacterium during infection. Additionally, the field needs higher-resolution analyses to identify specific residues and domains required for function to intelligently design future therapeutic and vaccine strategies.

### 3.1 The importance of adhesins in host-pathogen interactions

Historically, genetic manipulation of *Leptospira* has been difficult due to the limited genetic tools available and low rates of transformation. Recent advances are redefining the way we approach research on this genus (Bourhy et al., 2005; Picardeau, 2008; Murray et al., 2009; Alves et al., 2010; Aviat et al., 2010; Pappas et al., 2015; Pappas and Picardeau, 2015; Fernandes et al., 2021a,b,c, 2023a,b; Fernandes and Nascimento, 2022). Two approaches have been taken: heterologous expression of pathogen genes in a non-pathogen, or knockdown and knockouts in a pathogen.

Heterologous expression involves using a shuttle vector encoding a protein from a species different from its original source. In the case of *Leptospira*, genes from pathogenic species are cloned into a shuttle vector and expressed in a non-pathogen. Several proteins, including LIC11436, LIC11612, LIC11711, LIC12631, LIC13411, LigA, LigB, LMB216, and the mammalian cell entry protein (mce), have been shown to increase binding of the saprophyte to their respective ligands (Figueira et al., 2011; Pinne et al., 2012; Zhang et al., 2012; Toma et al., 2014; Kochi et al., 2020; Surdel et al., 2022a). This approach can be adapted for *in vivo* studies as well. A model of hematogenous dissemination, originally developed to measure bloodstream survival and tissue adhesion in *Borrelia burgdorferi* (Caine and Coburn, 2015), was adapted for use with *Leptospira* in 2022 (Surdel et al., 2022a). Surdel et al. (2022b) demonstrated that LIC13411, when produced in *L. biflexa*, was sufficient to enhance binding to host tissues, highlighting its potential role during infection. Pappas and Picardeau (2015) engineered a new shuttle vector that facilitates complementation and heterologous expression with increased success, making this strategy accessible for all laboratories (Picardeau, 2008). With this technology now widely successful, it is expected to become a standard baseline requirement for future publications identifying adhesins in *Leptospira* spp.

The classic method for demonstrating genetic requirements is through knockout experiments followed by complementation if there is a phenotype. In *Leptospira*, cloning difficulties have limited this approach. Early methods of creating knockouts relied on random transposon mutagenesis, a method typically used for screening (Bourhy et al., 2005; Murray et al., 2009). If a gene of interest were found to be interrupted by chance, this strain could then be used for additional studies such as validation of adhesins. One of the initial targeted methods used transcription activator-like effectors (TALEs) to knockdown genes of interest (Pappas and Picardeau, 2015). Pappas et al. (2015) employed TALEs to show that reducing both LigA and LigB production significantly decreased the virulence of *L. interrogans*, as measured by death and colonization (Pappas and Picardeau, 2015).

More recently, CRISPR/Cas technology has been adapted for use in *Leptospira* (Fernandes et al., 2021a,b,c, 2023a,b; Fernandes and Nascimento, 2022). Fernandes et al. (2021c, 2023b) used CRISPR/Cas to assess the *in vivo* requirements of several adhesins initially characterized *in vitro*, including LigAB, LipL32, LipL41, and LipL21. However, determining adhesin function with genetic mutants is challenging due to functional redundancy. For instance, only minimal changes were observed in the ability of these mutants to bind host molecules (Fernandes et al., 2021c, 2023b). Mutants of LigAB, LipL21, and LipL41 reduced death in hamsters, but leptospires still were able to colonize the kidneys (Fernandes et al., 2021c, 2023b). In contrast, a LipL32 mutant increased death in hamsters, potentially due to the altered proteome in this mutant (Fernandes et al., 2021c, 2023b). Another important consideration is the temporal requirements of each adhesin during infection. In *B. burgdorferi*, it is becoming increasingly clear that different adhesins are required for different steps of the endothelial transmigration process (Tan et al., 2023), and this could be true for various processes in *Leptospira* as well.

Advancements in genetic tools now enable testing of multiple mutations (Fernandes et al., 2023a), enhancing our ability to assign functions to proteins with redundant roles. Despite these strides, only a few adhesins have demonstrated importance *in vivo*, and none have been shown to be essential for virulence. With these genetic tools readily available, the adhesins previously identified *in vitro* can now be fully evaluated. This will be crucial in determining the role adhesins play in the pathogenesis of *Leptospira*.

### 3.2 Moving toward structure and function analysis

Understanding structure–function relationships of adhesins is essential for developing targeted vaccine and therapeutic strategies. By characterizing proteins at the sequence level, research can pinpoint regions that are critical for protein function. However, in *Leptospira*, there is currently very little information on candidate adhesin structure–function relationships. Eight complete structures of adhesins have been solved including Lp49 (Giuseppe et al., 2008), Lsa45 (Santos et al., 2023), LSS11580 (Hsu et al., 2020), LIC10831 (Miras et al., 2015), LIC11098 (Miras et al., 2015), LIC12759 (Miras et al., 2015), LIC12234 (Miras et al., 2015), and LipL32 (Hauk et al., 2009; Vivian et al., 2009; Tung et al., 2010). Two proteins, LigA (Mei et al., 2015a; Kumar et al., 2023) and LigB (Ptak et al., 2014; Mei et al., 2015b), only have specific domains solved.

Among these structures, only a couple have been investigated for structure–function relationships. Specific domains LigB, including LigB4 and LigB12, are important for binding human fibrinogen and tropoelastin (Hsieh et al., 2016, 2017). A few residues within LigB12 have been identified as important for binding, but a comprehensive structure–function analysis of LigB is still lacking (Hsieh et al., 2016, 2017). Mutation of residues within LipL32 that abolish calcium binding have shown that while calcium binding is not necessary for adhesion, it does influence the host immune response (Hauk et al., 2012; Lo et al., 2013).

Given this significant gap in knowledge, additional structure–function studies on leptospiral adhesins are crucial. This research is essential for designing vaccines and therapeutics targeting key epitopes recognized by the immune system. By separating distinct phenotypes in multi-functional adhesins, it may also be possible to engineer attenuated bacterial vaccines that are unable to bind and infect the host, while still displaying epitopes required to generate an adaptive immune response.

## 4 Conclusions

As new technologies continue to evolve in *Leptospira* research and other disciplines, methods for identifying and characterizing adhesins are also evolving. Techniques such as next-generation sequencing (NGS) offer more efficient characterization of genomic libraries than ever before. Experimental approaches that may have been under-used in the past, such as phage display or protein microarrays, may be combined with NGS to identify novel genes involved in host binding. These advancements are crucial for expanding our understanding of adhesin roles within a living host.

Using the new tools available in *Leptospira*, the field must now define which of the many candidates are in fact true adhesins. In the context of microbial pathogenesis, an adhesin must directly mediate the attachment of the bacteria to the host and be important during infection. Specifically, an adhesin must have a reasonable level of specificity and affinity (such as a K_D_ < 1 μM). It must contain a definable binding domain, where the structure of the protein-ligand interaction can be delineated. The gene must be expressed during infection, and inactivation of adhesin genes should lead to a binding and ultimately virulence defect (either alone, or in combination with proteins of redundant function). This defect must be due to the adhesin-ligand interaction specifically (as opposed to unrelated functions the protein may have during pathogenesis). As these candidates are evaluated using state-of-the-art approaches, the full arsenal of *Leptospira* adhesins will be defined and can then be targeted by future vaccine and therapeutic strategies.

Anti-adhesin therapy is a promising antibacterial strategy that can be used in conjunction with, or in lieu of, classical antibiotics. Every step of the adhesion process has been targeted, including modulating host receptors (Svensson et al., 1994, 2006), synthesizing peptides to compete for binding (Okuda et al., 2010; Huebinger et al., 2016), and immunization against adhesins (Cook et al., 2007; McNeilly et al., 2010). Anti-adhesin therapy has many advantages to typical antibiotics, including long term protection imparted by immunization. Krachler and Orth (2013) hypothesize that since anti-adhesin therapy does not affect the fitness of the organism, it is less likely to select for resistance. Disrupting adhesion in sites with high natural clearance rates (such as the kidney and bladder), will have the greatest impact. By targeting leptospiral adherence to the kidney proximal tubules, the bacteria will be forced into their planktonic state, thus increasing the rate of clearance through the natural route (Ternent et al., 2015).

A detailed understanding of the arsenal of adhesins used by *Leptospira* is needed. Overall, it is evident that the tools available for studying adhesin function in *Leptospira* spp. are advancing rapidly. With these technological advancements, future research on functions of adhesins must incorporate these tools to fully understand their relevance and roles in pathogenesis.
